# On the Non-Redundant Roles of TDO2 and IDO1

**DOI:** 10.3389/fimmu.2014.00522

**Published:** 2014-10-20

**Authors:** Paolo Puccetti

**Affiliations:** ^1^Department of Experimental Medicine, University of Perugia, Perugia, Italy

**Keywords:** TDO2, IDO1, AhR, indoleamine 2,3-dioxygenase, tryptophan

The *Perspective* by Pallotta et al. (1) offers the opportunity of a few more considerations on the mutually supportive relationship between tryptophan catabolic enzymes and the aryl hydrocarbon receptor (AhR) in the transitional response aimed at reinstalling homeostatic tolerance after meeting the needs of an acute inflammatory reaction (2). One major question is in fact – Why does AhR need two distinct sources of the ligand kynurenine to become activated, namely, tryptophan 2,3-dioxygenase (TDO2) and indoleamine 2,3-dioxygenase (IDO1) (3)? (The third known tryptophan catabolic enzyme, IDO2, has limited catalytic activity relative to the two others.).

The IDO1–AhR axis would, in fact, appear to be a highly efficient mechanism *per se*, in that it acts in a feedforward loop [i.e., kynurenine is an activating ligand for AhR, and activated AhR promotes *Ido1* transcription (4)], and it is self-regulated [AhR presides over regulatory proteolysis of IDO1, to restore homeostasis, as proposed by Pallotta et al. (1)]. For this loop to be operative, one need must be absolutely met, namely, the absence of IL-6, which operates its own mode of IDO1 proteasomal degradation via induction of SOCS3, resulting in the enzyme ubiquitination and proteolysis (5). Although it is one of AhR’s multiple jobs to transcriptionally repress *Il6* – through transcription of *Il10* (6) – the AhR–IDO1 system is IL-6-sensitive, and the “early” kynurenine leading to the recruitment of IL-10 to the transitional response must derive from a source other than the IDO1–AhR axis, that is, an enzyme not amenable to suppression by IL-6.

At the onset of an inflammatory response, IL-6 production is a necessary, defensive mechanism that need not be abrogated till the onset of the transition that will contribute to reinstall an optimal balance between inflammation and tolerance. Among the early events of systemic inflammation is cortisol-driven induction of acute phase proteins in liver. TDO2 is one such protein, and it is synthesized when production of proinflammatory cytokines, including IL-6, is at its height. *Tdo2* is not under AhR transcriptional control, nor is TDO2 amenable to IL-6-driven proteasomal degradation (3). This makes TDO2 production of kynurenine – and AhR activation – compatible with the events initiating the transitional response (Figure 1).

**Figure 1:**
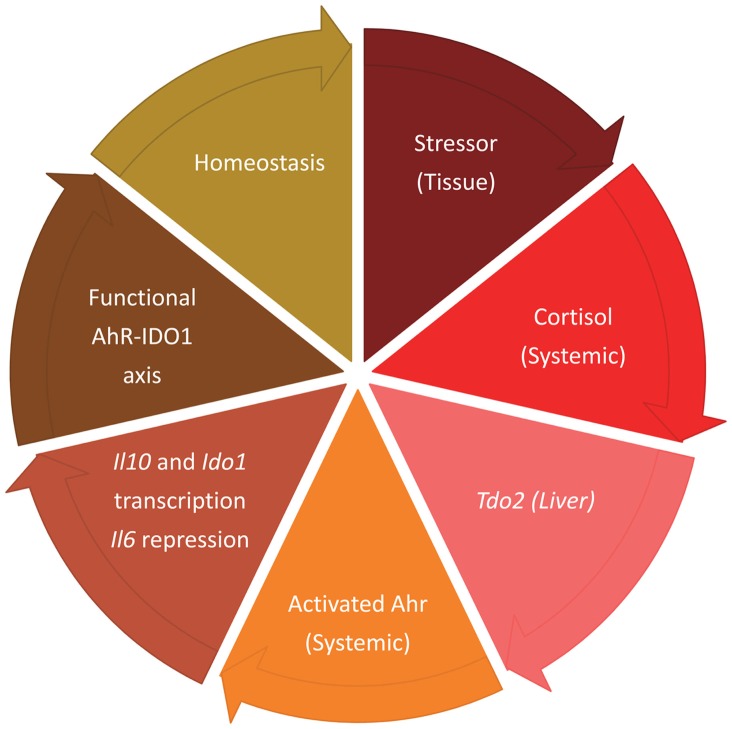
**Sequential events marking the transitional response aimed at reinstalling homeostatic tolerance after meeting the needs of an acute inflammatory reaction, with TDO2 and IDO1 having temporally segregated roles**.

The conclusion is that the roles of TDO2 and IDO1 as a source of AhR ligands are not redundant, the major differences lying in the different modalities of their respective gene activation, their being temporally segregated in function, and their disparate susceptibilities to inhibition by IL-6.

## Conflict of Interest Statement

The author declares that the research was conducted in the absence of any commercial or financial relationships that could be construed as a potential conflict of interest.
